# Study of Anti-Inflammatory and Analgesic Activity of Scorpion Toxins DKK-SP1/2 from Scorpion *Buthus martensii* Karsch (*Bm*K)

**DOI:** 10.3390/toxins13070498

**Published:** 2021-07-17

**Authors:** Yunxia Liu, Yan Li, Yuchen Zhu, Liping Zhang, Junyu Ji, Mingze Gui, Chunli Li, Yongbo Song

**Affiliations:** 1 College of Medical Devices, Shenyang Pharmaceutical University, 103 Wenhua Road, Shenyang 110016, China; liu15735177891@163.com (Y.L.); gui19970610@163.com (M.G.); 2 School of Life Science and Biopharmaceutics, Shenyang Pharmaceutical University, 103 Wenhua Road, Shenyang 110016, China; yueli_sy@163.com (Y.L.); zhuyuchen0421@gmail.com (Y.Z.); zlpstronger@163.com (L.Z.); jijunyu96@163.com (J.J.)

**Keywords:** *Buthus martensii* Karsch (*Bm*K), inflammatory, analgesia, DKK-SP1, DKK-SP2

## Abstract

*Buthus martensii* Karsch (*Bm*K), is a kind of traditional Chinese medicine, which has been used for a long history for the treatment of many diseases, such as inflammation, pain and cancer. In this study, DKK-SP1/2/3 genes were screened and extracted from the cDNA library of *Bm*K. The DKK-SP1/2/3 were expressed by using plasmid pSYPU-1b in *E. coli* BL21, and recombinant proteins were obtained by column chromatography. In the xylene-induced mouse ear swelling and carrageenan-induced rat paw swelling model, DKK-SP1 exerted a significant anti-inflammatory effect by inhibiting the expression of Nav1.8 channel. Meanwhile, the release of pro-inflammatory cytokines (COX-2, IL-6) was decreased significantly and the release of anti-inflammatory cytokines (IL-10) were elevated significantly. Moreover, DKK-SP1 could significantly decrease the Nav1.8 current in acutely isolated rat DRG neurons. In the acetic acid-writhing and ION-CCI model, DKK-SP2 displayed significant analgesic activity by inhibiting the expression of the Nav1.7 channel. Moreover, DKK-SP2 could significantly inhibit the Nav1.7 current in the hNav1.7-CHO cells.

## 1. Introduction

The scorpion is one of the oldest arthropods on earth, which has evolved for more than 400 million years [1]. To date, there are more than 2200 kinds of scorpions in the world [2,3]. Among them, *Buthus martensii* Karsch (*Bm*K) is a well-known traditional Chinese medicine for the treatment of inflammation [4], epilepsy [5], facial paralysis and cancer [6]. Scorpion venoms are secreted by the scorpion gland, which is classified as protein (scorpion toxins and different enzymes) and non-protein parts. Among them, scorpion toxins are believed as the major component for its pharmacological actions [7]. Until recently, only 400 out of 100,000 toxins were discovered in the scorpion gland [8]. Typically, the scorpion toxins have consisted of 28 to 76 amino acid residues with 3–4 pairs of disulfide bonds. The scorpion toxins could recognize and activate ion channels receptors so as to change their permeability. According to their combined receptors, scorpion toxins can be divided into Na^+^ channel toxins [9], K^+^ channel toxins [10], Cl^-^ channel toxins and Ca^2+^ channel toxins [11]. More specifically, Na^+^ channel toxins can be further divided into α-scorpion toxins and β-scorpion toxins [12]. α-scorpion toxins act on site 3 of the Na^+^ channel in a voltage-dependent manner and affect inactivation, while β-scorpion toxins act on site 4 of the Na^+^ channel in the same manner but affect activation [13].

Addiction, constipation, gastrointestinal ulcers and bleeding [14,15] are usually side effects for the commonly used clinical analgesics, such as morphine [16], codeine, ibuprofen [17], meloxicam, etc [18]. Recent studies have indicated that toxins from *Bm*K, such as *Bm*KIT2 [19], *Bm*KI1, *Bm*KI6, *Bm*KAS [20], *Bm*KAS-1, *Bm*KAGAP [21] had demonstrated excellent analgesic effects for long term use with no addictions [22,23] in the ION-CCI test, acetic acid-writhing and formalin analgesic model [12]. Meanwhile, toxins from *Bm*Kpp [24], *Bm*Kn2 [25], *Bm*Ka1 and *Bm*Ka2 were also found to exhibit potent anti-inflammatory activities. Therefore, it is promising to explore and identify these toxins from more biological sources.

Preparations of toxins from natural *Bm*K are tedious with high cost and lower yields. Several successful attempts to obtain scorpion toxins in *E.col*i [26], plants [27], yeast [28] and insect [29] expression systems were reported with mild yield and solubility. Since scorpion toxins are rich in disulfide bonds, there would be some hurdles to leap to achieve soluble expression in the prokaryotic system. Our previous efforts had been made to establish a series of vectors with high solubility recombinant protein expressions, the major improvements could be attributed to the co-expression of solubility-enhancing genes, such as N-Utilization substance (NusA), thioredoxin A (TrxA) and intein [30].

In this study, two novel scorpion toxins from *Bm*K were found and prepared in our *E. coli* expression systems. Their analgesic and anti-inflammatory biological activities and possible mechanism were elucidated, which could be used to further evaluate their druggability.

## 2. Results

### 2.1. mRNA Extraction, cDNA Synthesis, Target Gene Amplification and Sequencing Analysis

#### 2.1.1. mRNA Extraction, cDNA Synthesis and Target Gene Amplification

*Bm*K were stimulated for 24 h and their tails (0.3 g) were cut and ground in liquid nitrogen. The total RNA was extracted with the Trizol kit. The result is shown in Figure S1A. The cDNA target *Bm*K gene was amplified by using DKKSPF0/R0 as primers, and the PCR result is shown in Figure S1B.

**Figure 1:**
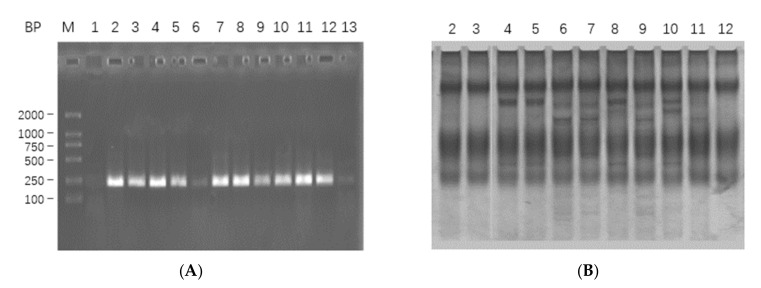
(**A**) The PCR analysis of 11 recombinants in 1.5% agarose electropheresis. Lane M: DL2000; Lane 1: PCR amplification of pSYPU-1b vector as negative control; Lanes 2–12: PCR amplification of 11 recombinants, indicating the fragment about 200 bp. (**B**) The screens analysis of PCR-SSCP on 10% SDS-PAGE for Lanes 2–12.

The PCR products were digested (*Nco* I and *Bam*H I) and inserted into plasmid pSYPU-1b, then recombinant plasmids were transformed into DH5α competent cells by standard protocols. There were 11 recombinants identified by PCR, and the results are shown in Figure 1A. PCR-SSCP electrophoresis (Figure 1B) and gene sequencing (Figure S2) results were used to screen and classify those identified recombinants. All these 11 recombinants could be classified into three genotypes, i.e., DKK-SP1 (lanes 2, 3 and 12); DKK-SP2 (lane 4, 5, 8 and 10) and DKK-SP3 (lane 6, 7, 9 and 11).

#### 2.1.2. Analysis of Sequencing Results

**Figure 2:**
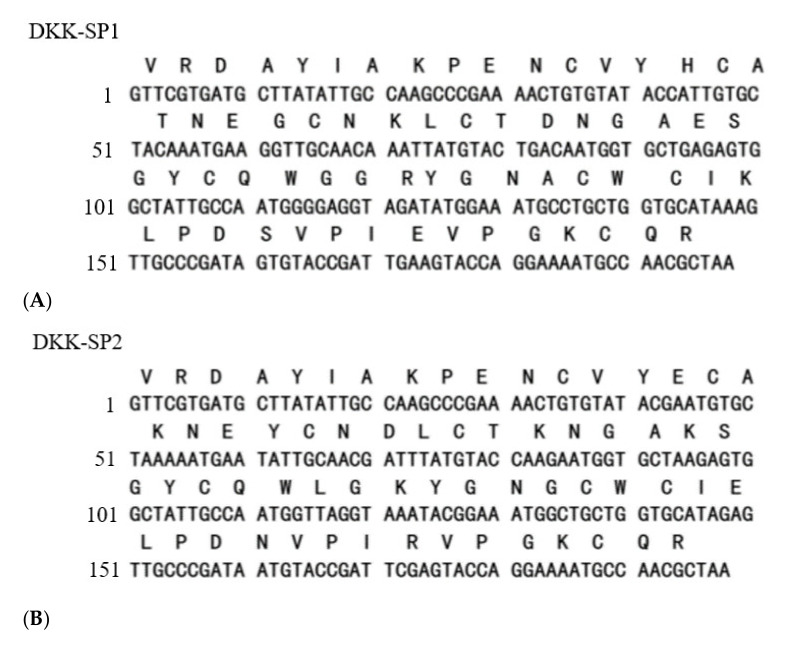

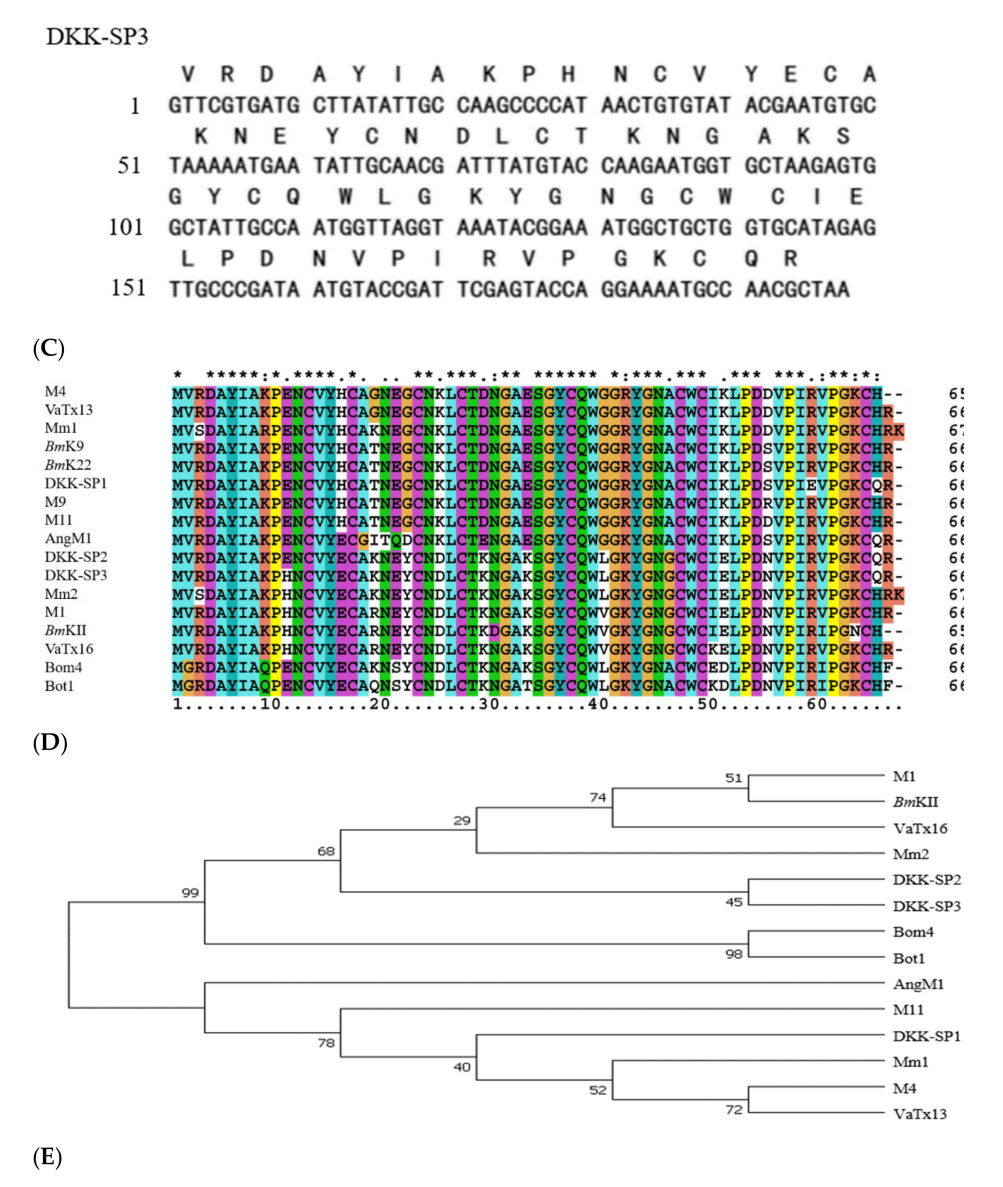
Nucleotide and translated amino acid sequences for DKK-SP1 (**A**), DKK-SP2 (**B**) and DKK-SP3 (**C**). (**D**) Multiple amino acid sequences alignment by CLUSTAL 2.0.12. (**E**) Unrooted phylogenetic tree of peptides from *Bm*K by Tree View.

The sequencing results showed that all three nucleotide sequences isolated from the *Bm*K cDNA were 198bp, encoding 66 amino acids. The gene sequences of lane3 (DKK-SP1), lane5 (DKK-SP2), and lane6 (DKK-SP3) are highly different (Figure 2A–C). By comparing amino acid sequences, it is indicated that DKK-SP1 has 92.19% identify with the scorpion alpha-like toxin *Bm*K M4(PDB ID:1SN4) [31,32], DKK-SP2 and DKK-SP3 has 93.75% and 95.31% identify with the scorpion alpha-like toxin *Bm*K M1 (PDB ID: 1DJT) (Figure 2D) [33]. Therefore, DKK-SP1, DKK-SP2, and DKK-SP3 could be assigned to α-scorpion toxins. Among these three peptides, the identity of a deduced amino acid between DKK-SP1 and DKK-SP2, DKK-SP1 and DKK-SP3 is 81.82% and 80.30%. There is only one amino acid difference between DKK-SP2 and DKK-SP3, with the identity of 98.48% (Sequences submitted to NCBI, waiting for accession No.).

### 2.2. Purification and Solubility Analysis of pSYPU-1b-DKK-SP1/SP2/SP3

**Figure 3:**
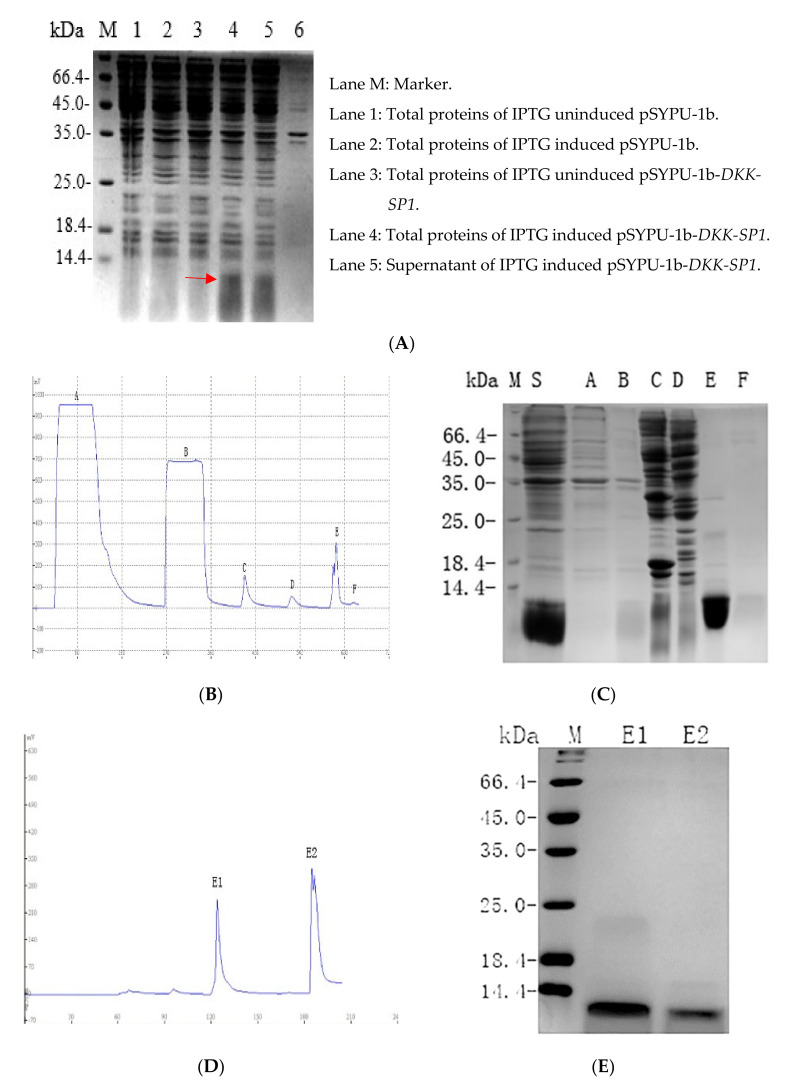
(**A**) 12.5% SDS-PAGE analysis of pSYPU-1b-*DKK-SP1* expressed in *E. coli* (DKK-SP1 is marked by a red arrow). (**B**) The protein profiles of Nickel chelation affinity chromatography. (**C**) 12.5% SDS-PAGE analysis of the elution fractions in (**B**) (Lane S: Sample. Lane A: fractions in permeate. Lane B-E: The corresponding peaks of buffer B, C, E. Lane F: Peak of EDTA. (**D**) The protein profiles of SP Sepharose High Performance chromatography. (**E**) 15% SDS-PAGE analysis of the elution fractions in (**D**). Lane M: Marker; Lane E1: Peak of buffer E1(TrxA); Lane E2: Peak of buffer E2(DKK-SP1).

In order to improve the purification and solubility of the recombinant protein, recombinant plasmid pSYPU-DKK-SP1/SP2/SP3 were used as templates and DKK-SPF1/R0 was used as primers to add the sequence encoding Tag-(His)_6_ at the N-terminus of DKK-SP1/SP2/SP3 by PCR. The PCR products were inserted into plasmid pSYPU-1b (with TrxA) by *Nco*I and *Bam*HI. The resultant pSYPU-1b-*DKK-SP1/SP2/SP3* vectors were then transformed into BL21. After induction by IPTG (37 °C, 4 h), 90% of the target protein was present in the supernatant with TrxA (Figure 3A). The nickel chelation affinity chromatography result was shown in Figure 3B. SDS-PAGE indicated the results containing DKK-SP1 and TrxA eluted with buffer E (Figure 3C). The elution fraction E peak was loaded to SP Sepharose High Performance chromatography to separate the solubilizing protein TrxA from the DKK-SP1 (Figure 3D). The SDS-PAGE results indicated that elution peak E1 contains auxiliary TrxA (11.8 kDa) that contributes to increase the productivity and solubility of peak E2 (DKK-SP1 (10 kDa)). Fraction E2 on the SP Sepharose High Performance chromatography is shown with RP-HPLC to be a single peak (Figure S3). The separation and purification results of DKK-SP2 and DKK-SP3 are shown in Figures S4 and S5, respectively.

### 2.3. Acute Toxicity on DKK-SP1/SP2/SP3

After the intravenous injection of DKK-SP1/SP2/SP3, the mortalities of mice were monitored for 14 days. The results were shown in Tables S1–S3. The LD_50_ value of DKK-SP1 was 20.57 mg/kg (95%CI, 18.09~23.1 4 mg/kg). The LD_50_ value of DKK-SP2 was 18.09 mg/kg (95%CI, 15.63~20.38 mg/kg). The LD_50_ value of DKK-SP3 was 4.31 mg/kg (95%CI, 3.63~5.00 mg/kg), indicating that the DKK-SP3 has high toxicity.

### 2.4. Research on Anti-Inflammatory Activity of DKK-SP1

#### 2.4.1. Anti-Inflammatory Activity of DKK-SP1

**Figure 4:**
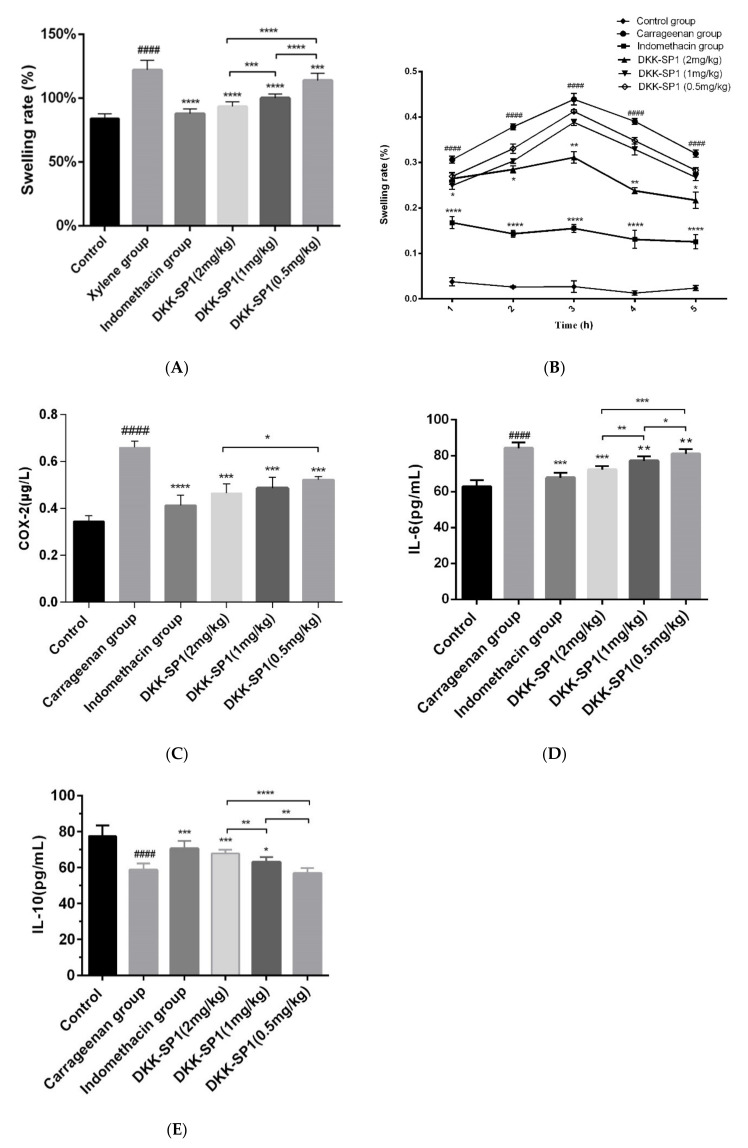
The inhibitory effect of DKK-SP1 on ear swelling in mice (**A**) and paw swelling in rats (**B**). (**C**) Effect of DKK-SP1 on the contents of COX-2 in the hind paw homogenates of carrageenan-induced rats. (**D**) Effect of DKK-SP1 on the contents of IL-6 in the hind paw homogenates. (**E**) Effect of DKK-SP1 on the contents of IL-10 in the homogenized hind paw. All data were presented as mean ± SEM. *n* = 12, #### *p* < 0.0001 compared with control group. * *p* < 0.05, ** *p* < 0.01, *** *p* < 0.001, **** *p* < 0.0001 compared with carrageenan group/xylene group.

As shown in Figure 4A, in the mice ear swelling model induced by xylene, compared with the control group, the ear swelling rate of the model group was significantly increased. In comparison with the model group, both indomethacin and DKK-SP1(2 mg/kg) significantly decreased the ear swelling rate. Although the effect of DKK-SP1 was not as strong as indomethacin, DKK-SP1 still was very effective in inhibiting swelling in a dose-dependent manner.

The anti-inflammatory effects of DKK-SP1 were also investigated in rats using the carrageenin-induced paw swelling model. The swelling rate of the paw was highest at 3h-4h, as shown in Figure 4B. Moreover, as compared to the model group, DKK-SP1 groups and indomethacin group could significantly decrease the paw swelling rate, and the effect of DKK-SP1 was in a dose-dependent manner.

#### 2.4.2. Effects of DKK-SP1 on Cytokine Secretion

The hind paw homogenates were used to detect the content of COX-2, IL-6 and IL-10 (Figure 4C–E). The results showed that the release of COX-2 and IL-6 in the carrageenan group were elevated significantly and the release of IL-10 was decreased significantly than those in the control group. In contrast, in comparison with the carrageenan group, the release of COX-2 and IL-6 were significantly decreased, and the release of IL-10 was significantly elevated in the DKK-SP1 groups and indomethacin group.

#### 2.4.3. Effects of DKK-SP1 on the Nav1.8 Expression and Current Density of Nav1.8

**Figure 5:**
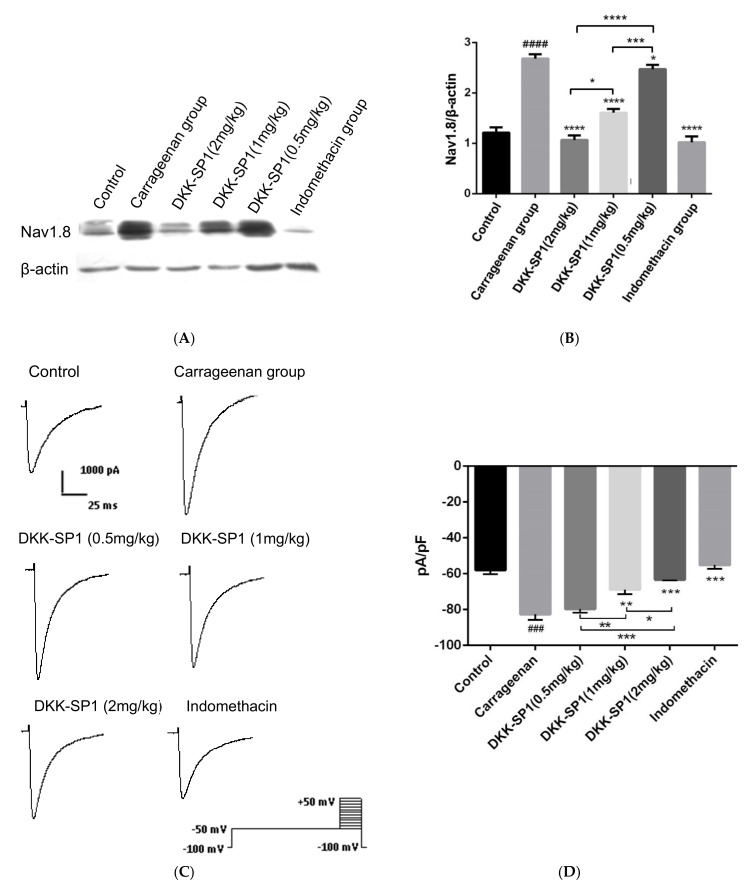
Change of Nav1.8 expression and currents in DRG neuron after carrageenan-induced paw edema model and DKK-SP1 injection in rats. (**A**,**B**) The expression of Nav1.8 in the DRG. (**C**) Nav1.8 currents were evoked by a depolarization voltage step from −50 mV to +40mV in 10mV increments from a holding potential of −100mV. (**D**) As compared to the carrageenan model group, both the indomethacin and DKK-SP1(2 mg/kg) inhibited the Nav1.8 current, and DKK-SP1 reducing the current density of Nav1.8 in a dose-dependent manner. Data were shown as mean ± SEM, *n* = 12. Compared with control group: ### *p* < 0.001, #### *p* < 0.0001. Compared with carrageenan group: * *p* < 0.05, ** *p* < 0.01, *** *p* < 0.001, **** *p* < 0.0001.

The DRG of rats in each group was taken to detect the expression of Nav1.8 protein, as seen in Figure 5A,B. The results showed that the expression of Nav1.8 protein in the carrageenan group was elevated significantly than those in the control group. In comparison with the carrageenan group, the expressions of Nav1.8 in the indomethacin group and DKK-SP1 groups were significantly decreased in a dose-dependent manner.

Changes of Nav1.8 currents in DRG neurons were detected after the carrageenan-induced paw edema model and DKK-SP1 injection. Compared with the control group, the peak sodium current density mediated by Nav1.8 was significantly increased by 42.61%. Compared with the carrageenan model group, Nav1.8 peak current density after DKK-SP1 treatment was significantly decreased in a dose-dependent way. Among them, 2 mg/kg DKK-SP1 inhibited Nav1.8 currents by 23.41%. Indomethacin inhibited Nav1.8 currents by 33.1%. (Figure 5C,D).

From the above analysis, it is concluded that DKK-SP1 has a good anti-inflammatory effect, which may be achieved by reducing the expression of Nav1.8 protein and inhibiting Nav1.8 current.

### 2.5. Research on Analgesic Activity of DKK-SP2

#### 2.5.1. Analgesic Activities of DKK-SP1/SP2

The effective responses were evaluated at various DKK-SP2 dose levels, and the results were shown in Table S4. The ED_50_ of DKK-SP2 was 1.04 mg/kg (95%CI, 0.78~1.26 mg/kg).

The mouse-twisting which is one of the classic peripheral pain models was used to test the analgesic effects of DKK-SP1/SP2, and results are shown in Table 1. Compared with the normal saline, the DKK-SP2 groups and morphine group displayed lower twisting times and significant analgesic effects (*p* < 0.01). The analgesic effect of DKK-SP2 was slightly lower than the morphine group. However, compared with the normal saline, the DKK-SP1 group (1 mg/kg) displayed lower analgesic effects.

#### 2.5.2. Effect of DKK-SP2 on Mechanical Pain Threshold and Thermal Pain Threshold

**Figure 6:**
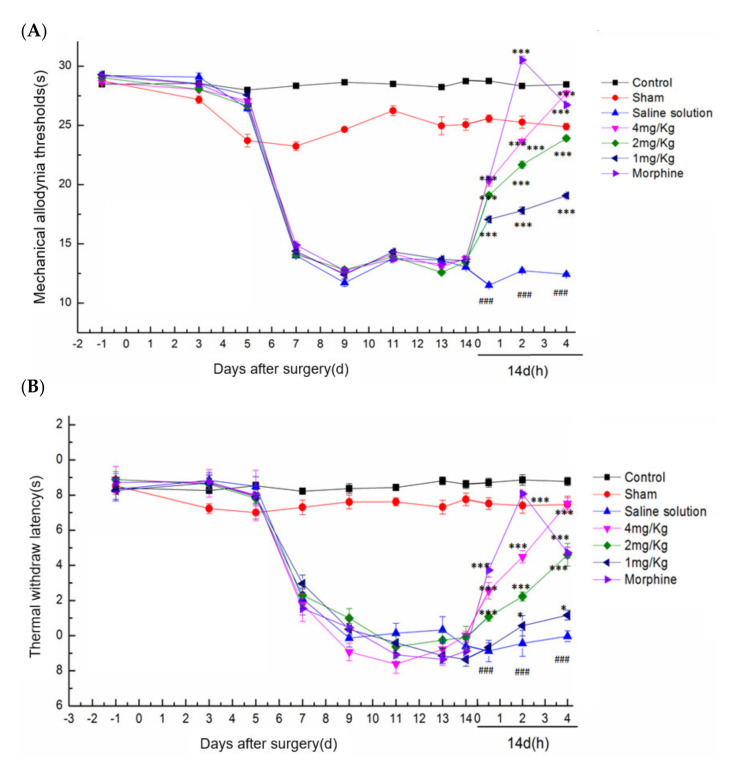
Effect of DKK-SP2 on mechanical allodynia thresholds and thermal withdraw latency in the rat ION-CCI model. (**A**) Effect of DKK-SP2 on the mechanical allodynia thresholds induced by ION-CCI surgery. (**B**) Effect of DKK-SP2 on the thermal withdrawal latency induced by ION-CCI surgery. All data were presented as mean ± SEM. *n* = 12. ### *p* < 0.001 compared with Sham group. * *p* < 0.05, *** *p* < 0.001 compared with saline solution group.

In the rat ION-CCI model, the analgesic effect of DKK-SP2 could be found in Figure 6A,B and Figure S6A,B. Compared with the sham operation group, the mechanical pain threshold and thermal pain threshold of the model group were significantly decreased, and the pain threshold reached the lowest after 14 days. Compared with the model group on 0.5, 2 and 4 h after administration, the DKK-SP2 groups displayed a significant increase in mechanical pain threshold and thermal pain threshold in the time-dependent and dose-dependent manner. The analgesic effect of DKK-SP2(4 mg/kg) for mechanical pain threshold was similar to that of morphine at 4 h. Meanwhile, for thermal pain threshold, that showed better analgesic effect than morphine at 4 h.

#### 2.5.3. Effects of DKK-SP2 on Nav1.7 Expression and Currents

**Figure 7:**
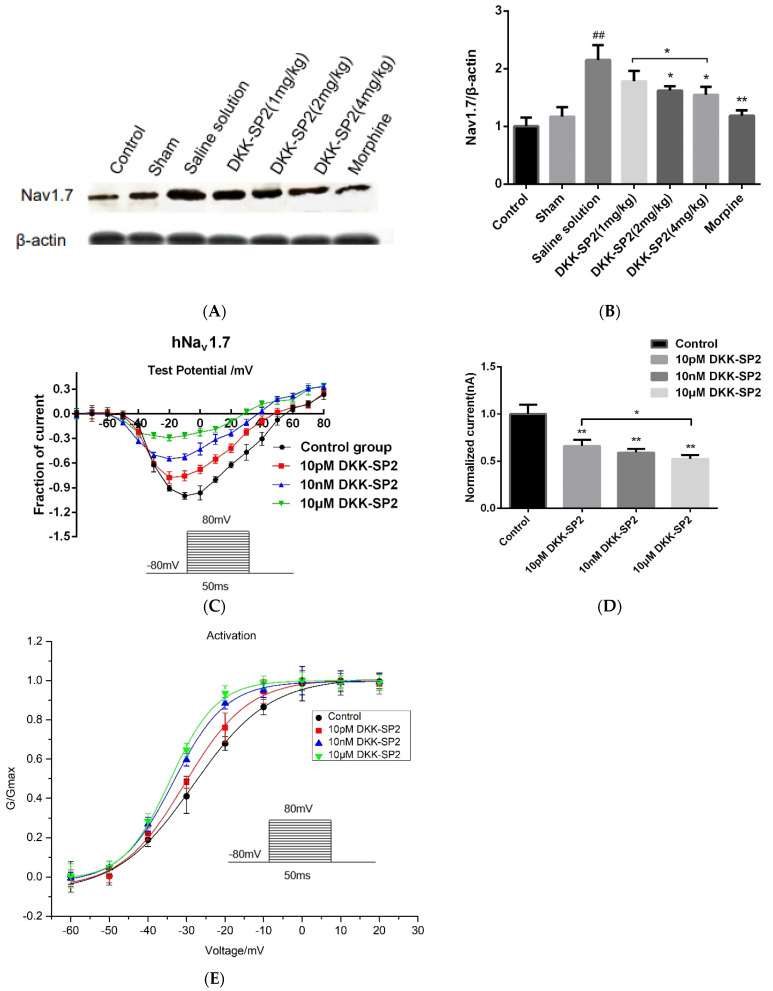
Effect of DKK-SP2 on Nav1.7 expression and Na^+^ peak current. (**A**,**B**) The expression of Nav1.7 in the TG. (**C**,**D**) The current-voltage relationship of hNav1.7 was evoked by DKK-SP2(10 pM, 10 nM, 10 μM). (**E**) Changes in hNav1.7 steady-state activation curves in hNav1.7-CHO after DKK-SP2(10 pM, 10 nM, 10 μM) treatment. Data were shown as mean ± SEM, *n* = 12. Compared with control group: ## *p* < 0.01. Compared with saline solution group/control group: * *p* < 0.05, ** *p* < 0.01.

The TG of rats in each group was taken to detect the changes of Nav1.7 expression (Figure 7A,B). Compared with the control group, the expression of Nav1.7 in the saline solution group was significantly increased. In comparison with the saline solution group, the expressions of Nav1.7 in the DKK-SP2 groups and morphine group were significantly reduced in dose-dependent manner.

Changes of Nav1.7 currents in hNav1.7-CHO cells were detected after pretreatment with the doses of DKK-SP2(10 pM, 10 nM, 10 μM). The results showed that DKK-SP2 significantly inhibited hNav1.7 in a dose-dependent manner, with inhibition rates of 66.37%, 59.33% and 52.70%, respectively (Figure 7C,D). The steady-state activation curve of the hNav1.7 current was shown in Figure 7E. Compared to the control group, the steady-state activation curve of the hNav1.7 current in the DKK-SP2 group shifted significantly in the direction of hyperpolarization and showed a dose-dependence. The V_1/2_ and slope factor *k* of the control group, 10 pM DKK-SP2, 10 nM DKK-SP2 and 10 μM DKK-SP2 group were −28.22 ± 1.00, 9.85 ± 0.74, −30.44 ± 0.77, 8.25 ± 0.92, −33.42 ± 0.40, 6.81 ± 0.36 and −34.24 ± 0.52, 6.04 ± 0.44, respectively.

According to the above analysis, DKK-SP2 has the analgesic effect, which may be achieved by reducing the expression of Nav1.7 protein and inhibiting Nav1.7 current.

## 3. Discussion

In this study, DKK-SP1, DKK-SP2 and DKK-SP3, which are composed of 66 amino acid residues, were first identified and screened from the *Bm*K cDNA library. The prokaryotic expression system pSYPU-1b was used, and its solubility-enhancing tag (TrxA) increased the solubility of the protein in E. *coli*. The protein was purified by nickel chelating affinity chromatography. After exploration, we found that when removing lipid and impurity protein, with 4% TritonX-100 and 30% ethanol elution, 20 column volumes, the removal effect is better, the purity of target protein is also improved.

**Figure 8:**
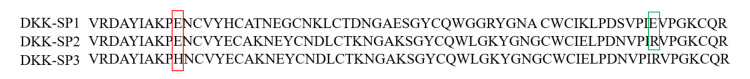
Primary structure diagrams of DKK-SP1, DKK-SP2 and DKK-SP3. the 10th amino acid residue is related to toxicity (red marked), the 58th amino acid residue is related to analgesia (green marked).

As it is known, the toxicity of scorpion toxins is a factor that must be considered before studying activity. The results showed that the LD_50_ of DKK-SP1(pI 5.91), DKK-SP2 (pI 6.32) and DKK-SP3 (pI 6.66) were 20.57 mg/kg (95%CI, 18.09~23.14 mg/kg), 18.09 mg/kg (95%CI, 15.63~20.38 mg/kg), 4.31 mg/kg (95%CI, 3.63~5.00 mg/kg), respectively. Since the toxicity of DKK-SP2 and DKK-SP3 was quite different, and only the 10th amino acids residue in the primary structure of protein was different, we preliminarily speculated that the 10th amino acid residue might be related to toxicity (Figure 8). From the literature that the pI value and the aromatic surface integrity of scorpion toxins are the most important factors affecting toxicity [32,33,34]. The smaller the pI value, the better the aromatic surface, the smaller the toxicity [33,35,36,37]. We preliminarily speculate that His10 (DKK-SP3), a basic amino acid, may affect the pI of scorpion toxins (increased DKK-SP3 pI), resulting in increased toxicity.

In the study of anti-inflammatory activity, the ear swelling and paw swelling belong to the acute inflammation models. They are characterized by simple operations and reach the peak of inflammation in a short time. Therefore, in this study, we took the ear swelling and paw swelling models as the research object to investigate the anti-inflammatory effect of DKK-SP1. From the experimental results, we found that DKK-SP1 can significantly decrease the ear swelling rate, paw swelling rate and the secretion of pro-inflammatory cytokines (IL-6, COX-2) in a dose-dependent manner. So we could get the conclusion that DKK-SP1 had a good anti-inflammatory effect.

When inflammation occurs in PNS, DRG will newly synthesize Nav1.8 protein and redistribute it to the distal parts and axons of the peripheral afferent nerve [38,39,40]. This phenomenon enhances the excitability and spontaneous ectopic discharge of small neurons in DRG and further enhances the inflammatory response [41]. Therefore, abnormally increased expression of Nav1.8 is considered to be a key factor in the formation of inflammation [42]. Furthermore, DKK-SP1 has 92.19% homology with the scorpion alpha-like toxin *Bm*K M4(PDB ID:1SN4). DKK-SP1 could be assigned to α-scorpion toxins which is a natural ligand for voltage-gated sodium channels. Therefore, we believe that the anti-inflammatory mechanism of DKK-SP1 may be related to the Nav1.8 channel. In this study, we took the paw swelling model as the research object to investigate the anti-inflammatory mechanism of DKK-SP1. From the experimental results, we found that after injection of carrageenan, the protein expression of Nav1.8 and the peak sodium current density mediated by Nav1.8 was significantly increased. Compared with carrageenan group, the protein expression of Nav1.8 and the peak sodium current density mediated by Nav1.8 was significantly decreased in the pre-administration group. It is concluded that DKK-SP1 has an anti-inflammatory effect, which is related to the Nav1.8 channel.

The analgesic activity of DKK-SP2 was studied by the ION-CCI of rats and the acetic acid writhing test of mice, which are commonly used animal models for preliminary study of analgesic activity. In the ION-CCI model, Nav1.7 is an important component of the formation of the ascending branch of action potential in TG. The expression level of Nav1.7 determines the degree of neuropathic pain [43,44,45]. Therefore, we studied the expression level of Nav1.7 in the ION-CCI analgesia model. The results showed that the expression of Nav1.7 in TG neurons was significantly up-regulated in the model group. When DKK-SP2 was administered, the expression of Nav1.7 was significantly decreased, and it was found in the patch-clamp experiment that DKK-SP2 could reduce the Nav1.7 current density in a dose-dependent manner, which preliminarily proved that the analgesic effect of DKK-SP2 was related to the Nav1.7 channel.

It has been reported that W38 Y42 R58 in scorpion venom plays a key role in the analgesic activity, especially R58 [46,47]. We found that the analgesic effect of DKK-SP2 (R58) was better than that of DKK-SP1 (E58) in the acetic acid-writhing test. Based on the fact that E58 is the acidic amino acid residue and R58 is the basic amino acid residue, we preliminarily speculated that the acid-base properties of the 58th amino acid residue may be an important factor leading to the difference in the activity of the two polypeptides. The verification of the site-directed mutagenesis experiment is still needed in the future.

## 4. Conclusions

In this study, three novel heter-expression toxin proteins were obtained from the *Bm*K scorpion toxin gland. DKK-SP1 exhibited a better anti-inflammation effect, which may be achieved by reducing the expression of Nav1.8 protein and inhibiting Nav1.8 current. DKK-SP2 displayed a better analgesic effect, which may be achieved by reducing the expression of Nav1.7 protein and inhibiting Nav1.7 current. DKK-SP3 was not further studied due to its greater toxicity. By analysis of the primary structure of the protein, we preliminarily speculate that the 10th amino acid residue plays a key role in toxicity and R58 is essential for analgesic activity. In the next step, more studies on pharmacological activities and the structure-function relationship should be performed to further elucidate the action mechanism of DKK-SP1/SP2.

## 5. Materials and Methods

### 5.1. Animal and Cell Cultures

*Buthusmartensii* Karsch (*Bm*K) were obtained from Shandong *Bm*K farm. Adult male Sprague Dawley rats weighing 150–180 g and adult Kunming mice (18–22 g) were used for studies. All animals were raised in the animal center of Shenyang Pharmaceutical University. All animal protocols were approved by the Institutional Animal Care and the regulations of the Animal Ethics Committee of Shenyang Pharmaceutical University, China ((SYXK (Liao) 2018-0009) which were approved on 21 December 2018).

hNa_v_1.7-CHO cells were cultured in IMDM (Gibco, Life Technologies, Inc., New York, USA) containing 10% FBS (Gibco, Life Technologies, Inc., New York, NY, USA), 16 μm thymidine, 2 mm GlutaMAX, 100 μm hypoxanthine, 100 U/mL penicillin/100 mg/mL streptomycin and 200 μg/mL G418 with 5% CO_2_ atmosphere at 37 °C.

### 5.2. Materials

Nucleic acid purification kits were purchased from Huashun Biological Engineering Co., Ltd., Shanghai, China. Restriction enzymes, PCR Ladder Marker, Taq DNA polymerase, pfu Taq, Buffer, and dNTPs were purchased from Nanjing Genescript Co.Ltd. Trizol kits were purchased from Invitrogen. Chelating Sepharose Fast Flow, SP Sepharose Fast Flow were purchased from GE Life Sciences. Indomethacin, carrageenan and xylene were purchased from Solarbio. Plasmid pSYPU-1b, *E. coli* strain DH5α, *E. coli* strain BL21, PVDF membrane (Merk Millipore, Billerica, MA), anti-Nav1.7 (1:500, Abcam), anti-Nav1.8 (1:500, Abcam), anti-β-actin (1:2000, Abcam), ELISA kits were purchased from Baolai Biotechnology (Jiangsu, China).

### 5.3. Gene Cloning and Sequencing

#### 5.3.1. cDNA Synthesis and Target Gene Amplification

*Bm*K were stimulated for 24 h and their tails (0.3 g) were cut and ground in the liquid nitrogen. Total RNA was extracted by Trizol kit according to the manufacturer’s instructions. cDNA first strand synthesis was performed by using Oligo(dT)17 as primers and total RNA as templates. The target genes were amplified using the complex of the cDNA first strand as a template. The amplification reactions were performed by primers DKKSPF0/R0, 10x pfu Taq PCR buffer, dNTP (2.5 mM), Pfu Taq (5 U/μL), and water. The PCR products were analyzed by 1.5% agarose electrophoresis.

Oligo(dT)17: 5′-CGGAATTCGGATCCGATATC (T)17(G/C/A)-3′
DKKSP F0: 5′-CATGCCATGGTTCGTGATGCTTATATTGCCAAG-3′
DKKSP R0: 5′-CGGGATCCTTAGCGTTGGCATTTTCCTGGTACT-3′

#### 5.3.2. Construction and Verification of the Expression pSYPU-1b-*DKK-SP1/SP2/SP3*

The PCR products were digested (*Nco*I and *Bam*HI) and inserted into plasmid pSYPU-1b, then recombinant plasmids were transformed into DH5α competent cells by standard protocols [30]. There were 11 recombinants colonies selected for PCR and SSCP analysis. The resultant three kinds of recombinants were classified as pSYPU-1b-*DKK-SP1/SP2/SP3*, respectively.

#### 5.3.3. Sequencing Analysis Softwares

The BLAST (http://www.ncbi.nlm.nih.gov/BLAST/, accessed on 15 January 2021) was used to search the nucleic acid and protein databases for identity analysis or similar sequences of target fragments. The CLUSTAL was used for nucleic acid sequence alignment, ClustaX1.83 was used for protein sequence alignment and Tree Viewer was used for systematic tree analysis.

### 5.4. Purification and Solubility of DKK-SP1 and DKK-SP2

In order to purify the target protein using the Nickel chelate affinity chromatography column, pSYPU-DKK-SP1/SP2/SP3 were used as templates and DKKSPF1/R0 was used as primers to amplify the target gene by PCR, in order to add *Nco*I and *Bam*HI restriction enzyme recognition sites at both ends and add the sequence encoding Tag-(His)_6_ at the N-terminus.

DKKSP R0: 5′-CGGGATCCTTAGCGTTGGCATTTTCCTGGTACT-3′
DKKSP F1: 5′-CATGCCATGGGACATCATCATCATCATCACGTTCGTGATGCTTATATTGCCAAG-3′

pSYPU-1b-*DKK-SP1/SP2/SP3* were inoculated in 3 mL LB (50 μg/mL kanamycin) and cultured at 37 °C for 4 h, OD_600_ value of 0.6–0.8. Then, IPTG (0.2 mmol/L) was added to induce expression for 4h at 37 °C. Bacteria were harvested at 3500 rpm for 20 min, washed twice with 40 mL of saline and resuspended in buffer A (pH8.0, 50 mM PBS). Bacteria were then sonicated and centrifugated at 12,000 rpm. The supernatant and precipitation were analyzed by 12.5% SDS-PAGE electrophoresis respectively.

The supernatant was purified with a nickel chelate affinity chromatography column (1.6 × 10 cm). The column was equilibrated with buffer A (pH 8.0, 50 mM PBS, 1.0 mL/min). Then the non-specific proteins and lipid were eluted with buffer B (4% TritonX-100/0.1 M PBS pH 8.0, 1.0 mL/min) and buffer C (30% ethanol/0.1 M PBS pH8.0, 1.0 mL/min). The peak E (DKK-SP1 and TrxA) was eluted with buffer E (50 mM imidazole, 1.0 mL/min) and the protein was collected.

The peak E was further purified with SP Sepharose High Performance chromatography (1.6 × 10 cm). The column was equilibrated with buffer G (50 mM citrate buffer, pH 3.6, 1.0 mL/min) and eluted by increasing the pH value (50 mM PBS, pH 6.0~pH 7.1, 1.0 mL/min) to remove the non-specific adsorption components. The target protein was eluted by changing salt ion concentration (pH 7.1, 0 M~1 M NaCl, 1.0 mL/min) and the protein was collected.

Peak E2 (DKK-SP1/SP2/SP3) was further purified by reversed-phase high-performance liquid chromatography (RP-HPLC) on TSK gel Protein C4-300 column (4.6 mm × 150 mm) using the solvents (A) 10% acetonitrile (containing 0.1% trifluoroacetic acid) and (B) 90% acetonitrile (containing 0.05% trifluoroacetic acid). A gradient elution was performed with a flow rate of 0.8 mL/min, starting with solvents (A) and finishing with solvents (B) over 50 min. The wavelength monitored was 280 nm.

### 5.5. Acute Toxicity Test

The LD_50_ of DKK-SP1/SP2/SP3 was determined in mice. The mice weighing 18–22 g, half male and half female, were randomly divided into groups (*n* = 12). Before administration, DKK-SP1/SP2/SP3 was diluted with normal saline. Mice were injected with DKK-SP1/SP2/SP3 of various concentrations or the same volume of normal saline through the tail vein. The mice were observed every 4 h on the first day after administration and then periodically thereafter, until the sacrifice of surviving animals after 14 days. During experiments, the mortalities of mice were monitored for 14 days.

The ED_50_ of DKK-SP2 was evaluated by the acetic acid-writhing. The mice with body weight of 18–22 g, half male and half female, were randomly divided into 12 mice in each group. Mice were injected with DKK-SP2 of various concentrations or the same volume of normal saline through the tail vein. After 15 min, 0.6% acetic acid (0.1 mL/10 g) was injected intraperitoneally. The time from the first twist of the mouse was calculated, and the duration of twists counting was 10 min. The inhibition rate of writhing reaction in each group was calculated.

Inhibition rate (%) = (number of writhing in control group − number of writhing in administration group)/number of writhing in control group × 100%.

### 5.6. Research on Anti-Inflammatory Activity of DKK-SP1

#### 5.6.1. Xylene-Induced Ear Edema Model

According to the previous protocol [48], the adult male mice were randomly divided into control group, xylene group, positive drug group (indomethacin), DKK-SP1 groups (2, 1, 0.5 mg/kg). DKK-SP1 and indomethacin were diluted with normal saline into each dose, with 12 mice in each group. The DKK-SP1 groups were intravenously injected DKK-SP1 (2, 1, 0.5 mg/kg) and the positive drug group was established by intraperitoneal injection of indomethacin (10 mg/kg). The control group and xylene group were intravenously injected with normal saline. One hour after the administration, each mouse was smeared with 30 μL xylene on the right ear, and the left ear was used as a control. Thirty minutes after inflammation induction [49], the mice were euthanized and both ears were taken by a 6 mm diameter symmetrical hole. The weight of the left and right ears was measured to calculate the mouse ear swelling rate (%).

Ear swelling rate (%) = (the right ear weight − the left ear weight)/the left ear weight × 100%

#### 5.6.2. Carrageenan-Induced Paw Edema Model

According to the previous protocol [50], the adult male rats were randomly divided into control group, carrageenan group, positive drug group (indomethacin), DKK-SP1 groups (2, 1, 0.5 mg/kg), with 12 rats in each group. DKK-SP1 and indomethacin were diluted with normal saline into each dose. The DKK-SP1 groups were intravenously injected DKK-SP1 (2, 1, 0.5 mg/kg) and the positive drug group was established by intraperitoneal injection of indomethacin (10 mg/kg). The control group and carrageenan group were intravenously injected with normal saline. After 1h, carrageenan (1%, 0.1 mL; except for the control group) was injected into the left hind paw pad. After injection of carrageenan for 0, 1, 2, 3, 4 and 5 h, the paw volumes were measured and recorded with a vernier caliper. After the final recording, the rats were anesthetized with ether and sacrificed, the hind paw and DRG were taken and stored in liquid nitrogen.

Paw swelling rate (%) = (paw volumes at each time point after carrageenan administration − paw volumes before administration)/paw volumes before administration) × 100%

#### 5.6.3. Enzyme-Linked Immunosorbent Assays

Paw tissues and PBS solution are poured into the homogenizer for grinding. After homogenizing, the supernatants were collected by centrifugation (12,000 rpm for 15 min at 4 °C). According to the ELISA manufacturer’s instructions, the levels of COX-2, IL-6 and IL-10 were measured.

### 5.7. Research on Analgesic Activity of DKK-SP2

#### 5.7.1. Acetic Acid-Writhing Test

The analgesic activity of DKK-SP2 was evaluated by the acetic acid writhing. The mice (male, 18–22 g) were randomly divided into control group, model group, morphine group (1.0 mg/kg), and DKK-SP2 groups (2, 1, 0.5 mg/kg), with 12 mice in each group. DKK-SP2 and morphine were diluted with normal saline. The mice were injected intravenously with normal saline, morphine, DKK-SP2 (2, 1, 0.5 mg/kg), respectively. Twenty minutes later, the mice were intraperitoneally injected with 0.6% (*w/w*) glacial acetic acid solution (0.1 mL/10 g) [51]. The time from the first twist of the mouse was calculated, and the duration of twists counting was 10 min. The persons conducting each of the behavioral tests were performed in a blinded manner.

#### 5.7.2. The Trigeminal Neuralgia Model (ION-CCI)

The adult male rats were randomly divided into control group, sham operation group, model group, DKK-SP2 groups (4, 2, 1 mg/kg), morphine group (1.0 mg/kg), with 12 rats in each group. DKK-SP2 and morphine were diluted with normal saline. SD rats were injected intraperitoneally with 4% chloral hydrate and fixed supine on the operating table after anesthesia. According to the previous protocol [52], the ION-CCI model was established. At 14 days after ION-CCI, DKK-SP2 groups were injected with the corresponding dose of scorpion toxins into the tail vein. The sham operation group and model group were injected with the normal saline and the morphine group was injected with morphine in the tail vein. Then, within 2 weeks after ION-CCI, mechanical stimulation and thermal radiation stimulation were applied. The persons conducting each of the behavioral tests were performed in a blinded manner.

In this experiment, the mechanical allodynia threshold of rats was measured. The rats were trained adaptively three days before modeling which were gently touched and stimulated with a pain measuring instrument. The mechanical allodynia threshold of the mouth and face of rats were tested by von-Frey electronic pain meter. The experiment recorded the intensity of withdrawal reaction (the head quickly dodge or blink), escape or scratching of the facial area, which was called mechanical pain threshold.

Moreover, the thermal withdrawal latency of rats was measured by a thermal pain meter, in which the temperature of the thermal radiation beam was 50 °C and the light source away from the measured area of the mouth and face was 8 cm. The rats were trained adaptively three days before modeling, the latent period of thermal stimulation on the day before modeling was recorded as the basic level of the test. The thermal withdrawal latency was recorded within 14 days after the rat face was irradiated with a focused beam of intensely radiant light. The cut-off time was 25 s.

At the end of the experiment, the rats were sacrificed, TG was taken and stored in liquid nitrogen.

### 5.8. Western Blotting

Total proteins of DRG from carrageenan-induced paw edema model and proteins of TG from the trigeminal neuralgia model (ION-CCI) were extracted with a protein extraction kit according to the manufacturer’s instructions. The extracted proteins were separated by SDS-PAGE, and then transferred to the PVDF membrane. The proteins were blocked with 5% skimmed milk for 2 h and incubated with indicated primary and secondary antibodies. After washing, Image Lab software was used to collect signals.

### 5.9. Electrophysiology

Rats were anesthetized by intraperitoneal injection of 3.5% chloral hydrate and DRG neurons were acutely isolated. The culture dish was placed in a 37 °C incubator, waiting for the cells to adhere to the wall, and whole-cell patch-clamp recording was performed 2–3 h later. The internal solution contained: CsCl (140 mM), TEA-Cl (10 mM), EGTA (10 mM), HEPES (10 mM), pH = 7.2. The external solution contained (in mM): NaCl (140 mM), MgCl_2_ (1 mM), CaCl_2_ (3 mM), KCl (5 mM), TEA-Cl (10 mM), 4-AP (1 mM), CdCl2 (0.2 mM), HEPES (10 mM), glucose (10 mM), pH = 7.3. Small-diameter DRG neurons were selected and given step voltage stimulation according to relevant protocols [53]. Currents were recorded by using an AxoPatch 200B amplifier (Axon Instruments, Foster City, CA, USA), and the signal was filtered and converted into data, which was then analyzed on the computer.

Whole-cell voltage-clamp recordings of hNav1.7-CHO were performed with the automated electrophysiology platform (NPC-1 and Port-a-patch system, Nanion Technologies GmbH, Munich, Germany) and an EPC10 Amplifier (HEKA Elektronik, Lambrecht, Germany). The internal and external solutions are the same as those used for conventional patch clamp experiments [54].

## 6. Data Analysis

All data were processed by SPSS26.0, Origin Pro9, Clampex10.0, Clampfit10.0 software and GraphPad Prism 7.0 statistical analysis software. LD_50_, ED_50_ and 95% confidence interval(CI) calculated by Bliss method. The voltage-clamp data were digitized and analyzed using pCLAMP10.2 software (Axon Instruments). *p* < 0.05 (*), *p* < 0.01 (**), *p* < 0.001 (***), *p* < 0.0001 (****), *p* < 0.01 (##), *p* < 0.001 (###) and *p* < 0.0001 (####) were considered statistically significant.

## Figures and Tables

**Table 1:** Effect of DKK-SP1/SP2 on the acetic acid-writhing test.

Groups	Dose (mg/kg)	Number of Writhes (Mean±SEM)	Inhibition Efficiency (%) ^1^
NS	-	36.5 ± 3.7	-
Morphine	1.0	8.1 ± 2.4 ***	77.81
DKK-SP2 (2 mg/kg)	2.0	8.5 ± 3.1 ***	76.71
DKK-SP2 (1 mg/kg)	1.0	13.6 ± 3.4 **	62.85
DKK-SP2 (0.5 mg/kg)	0.5	30.4 ± 4.8	16.71
DKK-SP1	1.0	31.7 ± 2.5	13.15

^1^ The inhibition efficiency is the ratio (T0−T)/T0, where T0 is the mean number of writhes in the negative control group and T is the mean number of writhes in the experiment groups with DKK-SP1/DKK-SP2 (*n* = 12). All data were presented as mean ± SEM, ** *p* < 0.01, *** *p* < 0.001 vs. normal saline.

## Data Availability

Data is contained within the article or Supplementary Material.

## Supplementary Materials

The following are available online at https://www.mdpi.com/article/10.3390/toxins13070498/s1, Figure S1: The RNA extraction and cDNA cloning. Figure S2: The results of gene sequencing. Figure S3: RP-HPLC of DKK-SP1 on a TSK gel Protein C4-300 column. Figure S4: Purification and solubility analysis of pSYPU-1b-*DKK-SP2*. Figure S5: Purification and solubility analysis of pSYPU-1b-*DKK-SP3*. Figure S6: The effects of DKK-SP2 on mechanical allodynia thresholds and thermal withdraw latency were in a dose-dependent manner in a rat ION-CCI model. Table S1: Effect of DKK-SP1 on mortality of mice. Table S2: Effect of DKK-SP2 on mortality of mice. Table S3: Effect of DKK-SP3 on mortality of mice. Table S4: Effect of DKK-SP2 on acetic acid writhing test.
