# Supplementary material for: Study of Anti-Inflammatory and Analgesic Activity of Scorpion Toxins DKK-SP1/2 from Scorpion Buthus martensii Karsch (BmK)
Source: Toxins (Basel). 2021 Jul 17;13(7):498. doi: 10.3390/toxins13070498 (PMC8310270; doi:10.3390/toxins13070498)
Supplement: Supplementary file 1 [file toxins-13-00498-s001.zip › toxins-1259465-supplementary.pdf]

Supplementary Materials: Study of Anti-Inflammatory and Analgesic Activity of Scorpion Toxins DKK-SP1/2 from Scorpion *Buthus martensii* Karsch (*Bm*K)

Yunxia Liu, Yan Li, Yuchen Zhu, Liping Zhang, Junyu Ji, Mingze Gui, Chunli Li and Yongbo Song

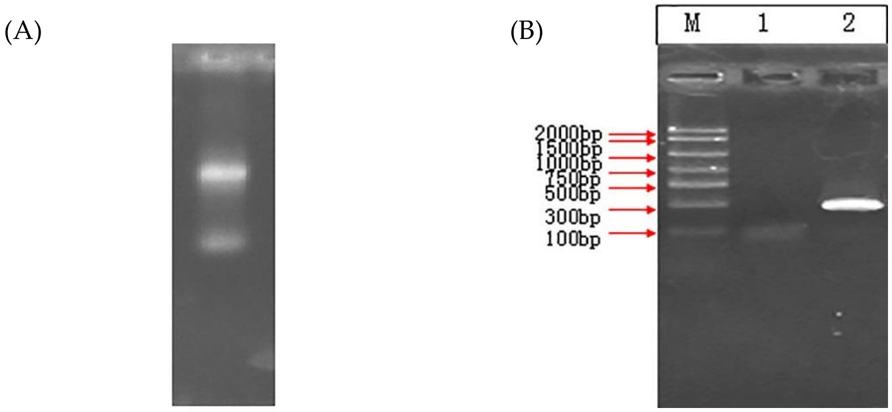

Figure S1. The RNA extraction and cDNA cloning. (**A**) Total RNA from scorpion tail. (**B**) The cDNA target gene of *Bm*K by 1.5% agarose gel. Lane M: DL2000; Lane1: The negative as control(water); Lane2: The double-stranded target cDNA.

(A)

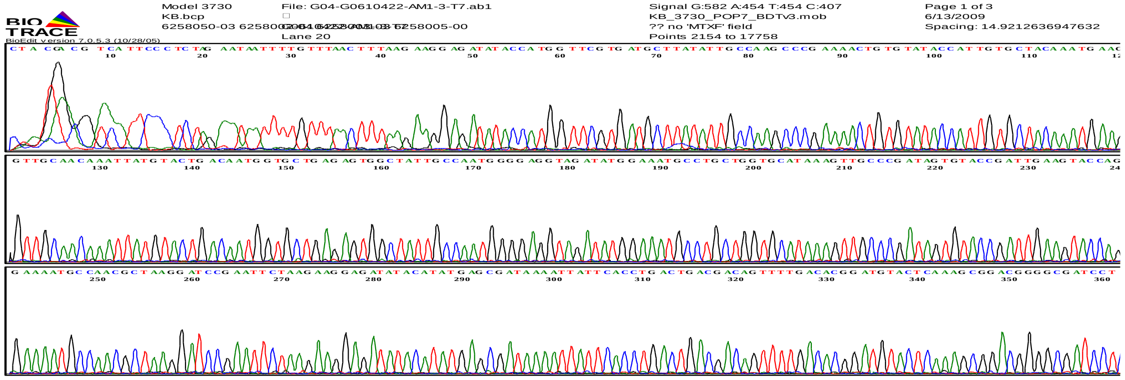

(B)

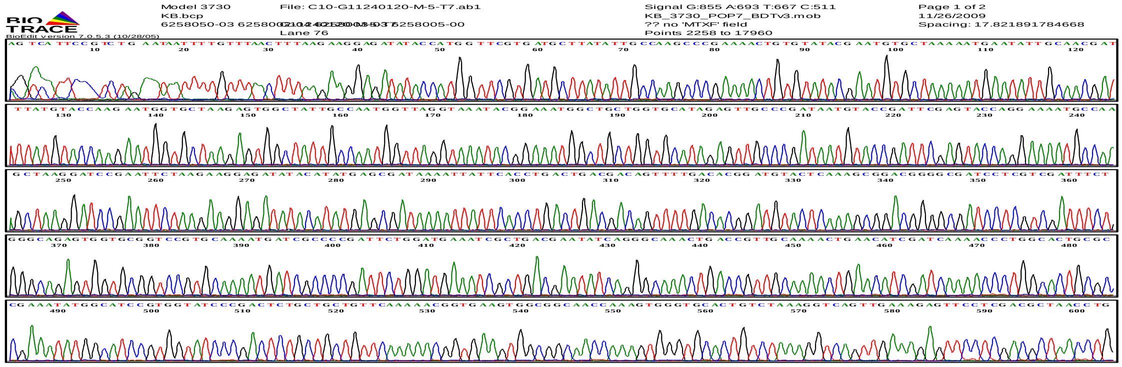

(C)

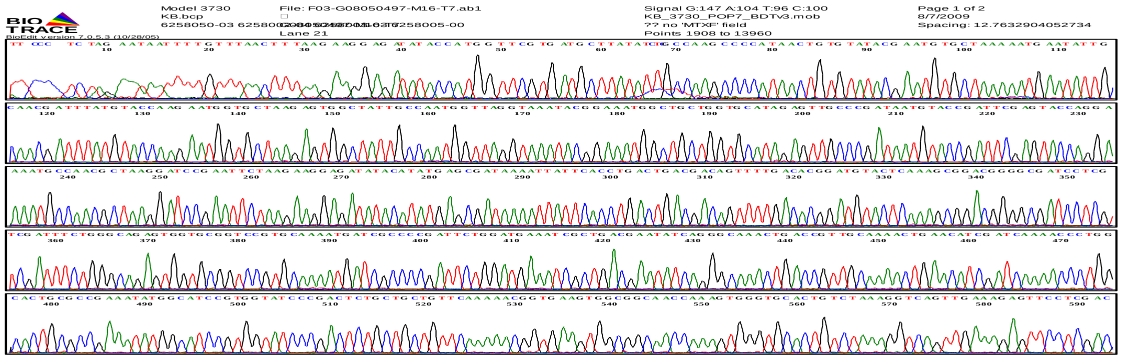

Figure S2. The results of gene sequencing. (**A**) The sequence of Lane 2,3,12. (**B**) The sequence of Lane 4,5,8,10. (**C**) The sequence of Lane 6,7,9,11.

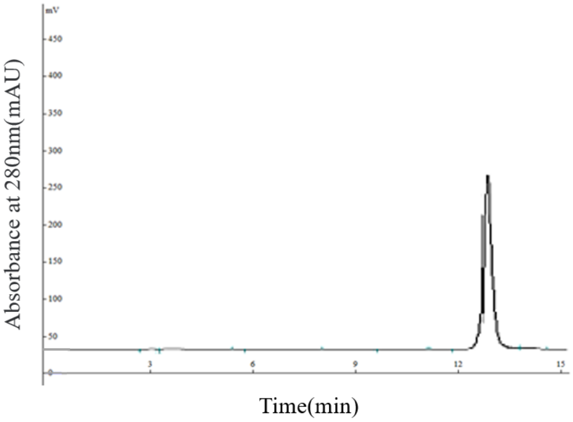

Figure S3. RP-HPLC of DKK-SP1 on a TSK gel Protein C4-300 column.

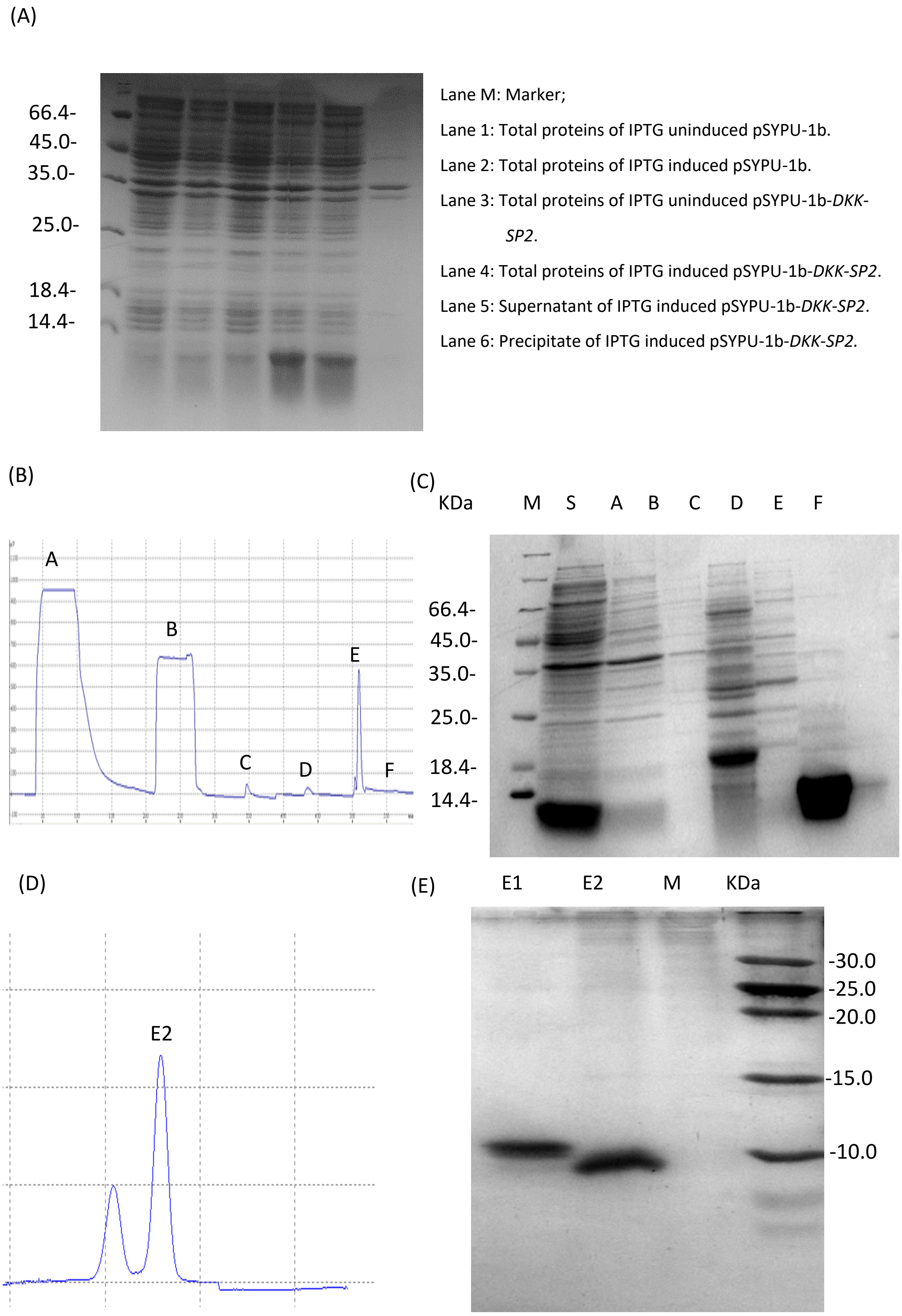
(A)
66.4-
45.0-
35.0-
25.0-
18.4-
14.4-
Lane M: Marker;
Lane 1: Total proteins of IPTG uninduced pSYPU-1b.
Lane 2: Total proteins of IPTG induced pSYPU-1b.
Lane 3: Total proteins of IPTG uninduced pSYPU-1b-*DKK-SP2*.
Lane 4: Total proteins of IPTG induced pSYPU-1b-*DKK-SP2*.
Lane 5: Supernatant of IPTG induced pSYPU-1b-*DKK-SP2*.
Lane 6: Precipitate of IPTG induced pSYPU-1b-*DKK-SP2.*
(B)
A
B
C
D
E
F
(C)
KDa
M S A B C D E F
66.4-
45.0-
35.0-
25.0-
18.4-
14.4-
(D)
E2
(E)
E1 E2 M KDa
-30.0
-25.0
-20.0
-15.0
-10.0

Figure S4. (**A**) 12.5% SDS-PAGE analysis of pSYPU-1b-*DKK-SP2* expressed in *E. coli*. (**B**) The protein profiles of Nickel chelation affinity chromatography. (**C**) 12.5% SDS-PAGE analysis of the elution fractions in Figure S4 (B) (Lane S: Sample. Lane A: fractions in permeate. Lane B-E: the corresponding peaks of buffer B, C, E. Lane F: peak of EDTA. (**D**) The protein profiles of SP Sepharose High Performance chromatography. (E) 15% SDS-PAGE analysis of the elution fractions in Figure S4 (D). Lane M: Marker; Lane E1: peak of buffer E1(TrxA); Lane E2: peak of buffer E2(DKK-SP2).

(A)

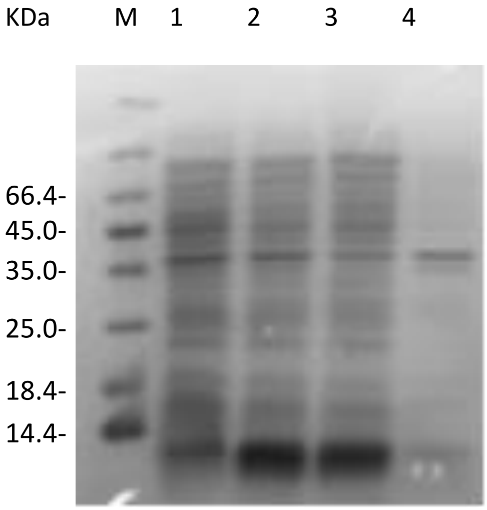

Lane M: Marker;

Lane 1: Total proteins of IPTG uninduced pSYPU-1b-*DKK-SP3.*

Lane 2: Total proteins of IPTG induced pSYPU-1b-*DKK-SP3*.

Lane 3: Supernatant of IPTG induced pSYPU-1b-*DKK-SP3*.

Lane 4: Precipitate of IPTG induced pSYPU-1b-*DKK-SP3*.

(B)

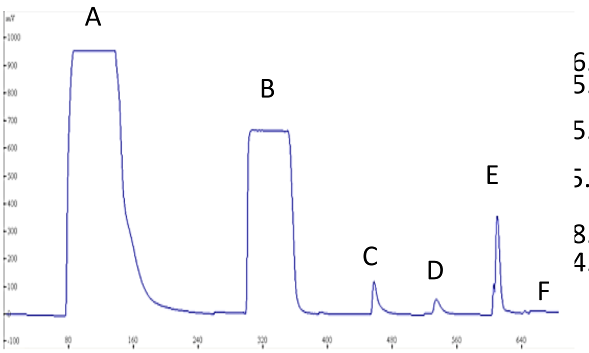

(C)

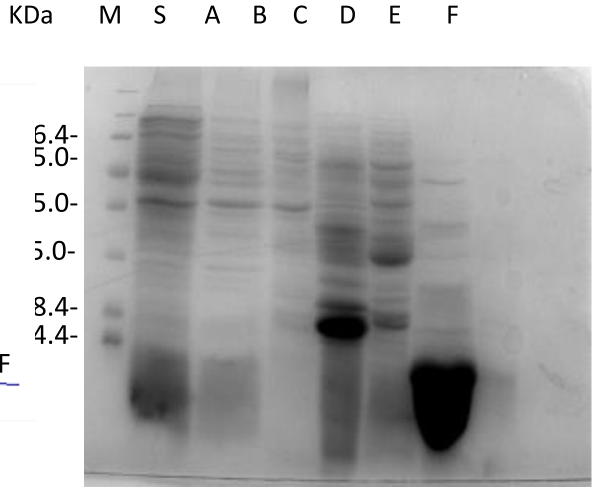

(D)

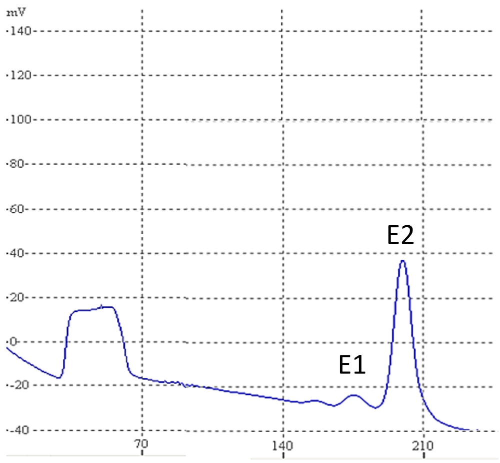

(E)

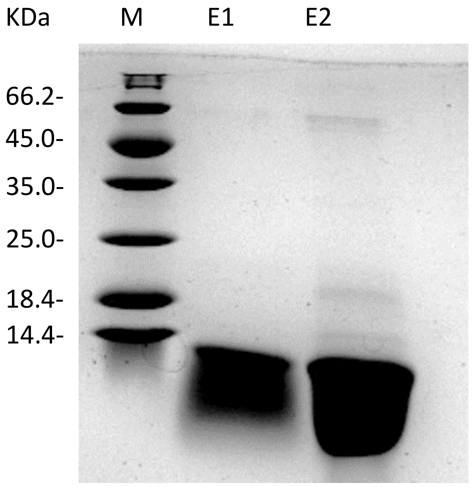

Figure S5. (**A**) 12.5% SDS-PAGE analysis of pSYPU-1b-*DKK-SP3* expressed in *E. coli*. (**B**) The protein profiles of Nickel chelation affinity chromatography. (**C**) 12.5% SDS-PAGE analysis of the elution fractions in Figure S5(B) (Lane S: Sample. Lane A: fractions in permeate. Lane B-E: the corresponding peaks of buffer B, C, E. Lane F: peak of EDTA. (**D**) The protein profiles of SP Sepharose High Performance chromatography. (**E**) 15% SDS-PAGE analysis of the elution fractions in Figure S5(D). Lane M: Marker; Lane E1: peak of buffer E1(TrxA); Lane E2: peak of buffer E2(DKK-SP3).

(A)

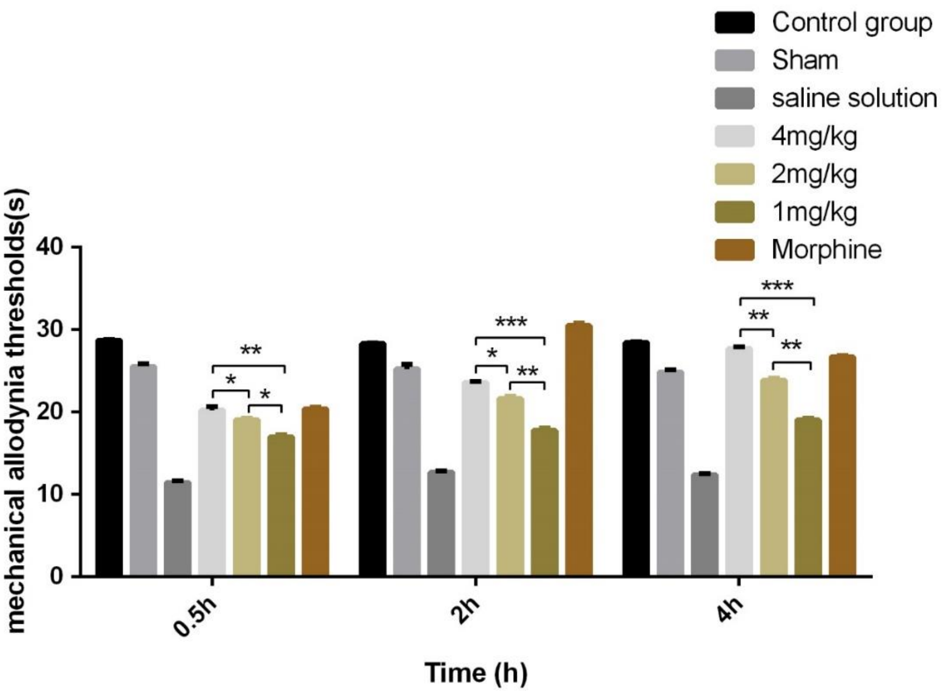

(B)

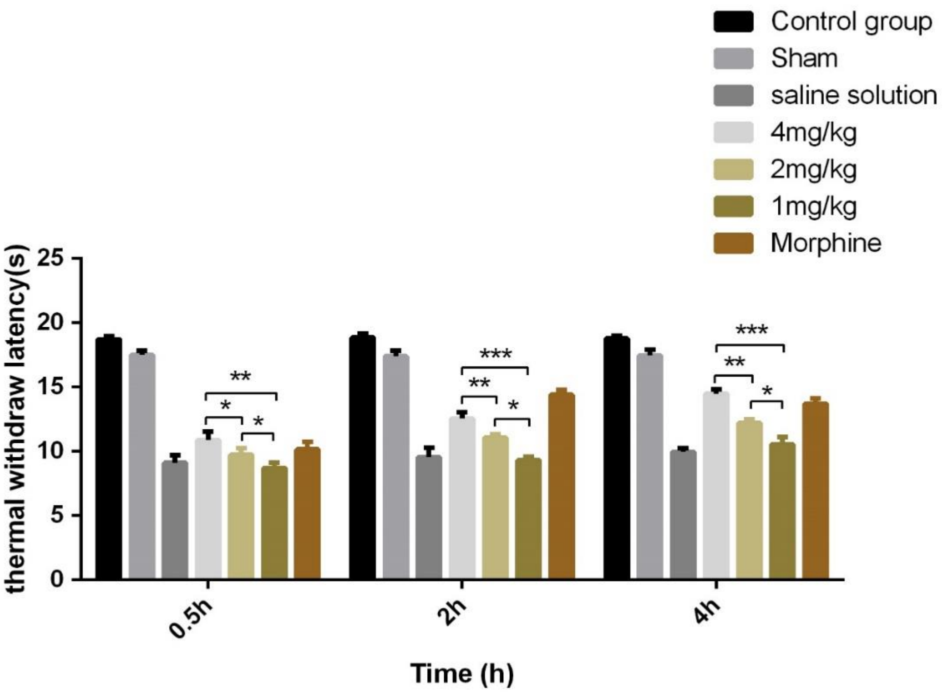

Figure S6. The effects of DKK-SP2 on mechanical allodynia thresholds and thermal withdraw latency were in a dose-dependent manner in a rat ION-CCI model (**A**) Effect of DKK-SP2 on the mechanical allodynia thresholds was in a dose-dependent manner. (**B**) Effect of DKK-SP2 on the thermal withdraw latency was in a dose-

dependent manner. All data was presented as mean ± SEM. $n = 12$, * $p < 0.05$, ** $p < 0.01$, *** $p < 0.001$.

Table S1. Effect of DKK-SP1 on mortality of mice.

Dose (mg/kg)	Total Mice	Dead Mice	Individual Survival Time (Days) (Mean Survival Time ± SEM)	Mortality %
NS	12	0	14	0
10	12	0	14	0
15	12	2	10, 12, 14, 14, 14, 14, 14, 14, 14, 14, 14, 14 (13.67±0.72)	17
20	12	5	7, 8, 10, 11, 12, 14, 14, 14, 14, 14, 14, 14 (12.17±6.14)	42
25	12	8	5, 6, 6, 7, 8, 10, 11, 12, 14, 14, 14, 14 (10.08±11.58)	67
30	12	12	3, 3, 4, 5, 6, 7, 8, 9, 9, 11, 12, 13 (7.50±10.75)	100

After the intravenous injection of DKK-SP1, the mortality of mice was monitored for 14 days. The results were shown in Table S1 and the LD_{50} values was 20.57mg/kg (95%CI, 18.09~23.14mg/kg) by Bliss method.

Table S2. Effect of DKK-SP2 on mortality of mice.

Dose (mg/kg)	Total Mice	Dead Mice	Individual Survival Time (Days) (Mean Survival Time ± SEM)	Mortality %
NS	12	0	14	0
10	12	0	14	0
15	12	4	6, 8, 10, 13, 14, 14, 14, 14, 14, 14, 14, 14 (12.42±7.24)	33
20	12	7	7, 9, 10, 10,11, 12, 13, 14, 14, 14, 14, 14 (11.83±5.31)	58
25	12	10	4, 4, 6, 8, 8, 10, 11, 11, 13, 13, 14, 14 (9.67±12.22)	83
30	12	12	2, 3, 5, 5, 6, 7, 7, 9, 9, 10, 12, 13 (7.33±10.56)	100

After the intravenous injection of DKK-SP2, the mortality of mice was monitored for 14 days. The results were shown in Table S2 and the LD_{50} values was 18.09 mg/kg (95%CI, 15.63~20.38mg/kg) by Bliss method.

Table S3. Effect of DKK-SP3 on mortality of mice.

Dose (mg/kg)	Total Mice	Dead Mice	Individual Survival Time (Days) (Mean Survival Time ± SEM)	Mortality %
NS	12	0	14	0
0.7	12	0	14	0
2.1	12	2	9, 11, 14, 14, 14, 14, 14, 14, 14, 14, 14, 14 (13.33±2.39)	17
3.5	12	4	7, 9, 11, 12, 14, 14, 14, 14, 14, 14, 14, 14 (12.58±5.24)	33
4.9	12	9	3, 5, 6, 7, 9, 10, 11, 13, 13, 14, 14, 14 (9.92±13.91)	75
6.3	12	12	2, 3, 3, 4, 5, 5, 6, 8, 8, 9, 11, 12 (6.33±9.72)	100

After the intravenous injection of DKK-SP3, the mortality of mice was monitored for 14 days. The results were shown in Table S3 and the LD_{50} values was 4.31mg/kg (95%CI, 3.63~5.00mg/kg) by Bliss method.

Table S4. Effect of DKK-SP2 on acetic acid writhing test.

Group	Dose/mg·kg$_{-1}$	Writhing times	Inhibition rate/%
NS	--	38.4±2.0	--
DKK-SP2	0.2	39.0±2.1	0.0
	0.8	25.5±2.8*	33.6
	1.4	10.1±3.7**	73.7
	2.0	8.2±3.4***	78.6
	2.6	1.6±2.3***	95.8
	3.2	0.0±0.0	100.0

The results are shown in Table S4, from which it can be measured that the half effective dose (ED_{50}) of DKK-SP2 is 1.04mg/kg (95%CI, 0.78~1.26mg/kg) by Bliss method. All data was presented as mean ± SEM. n = 12, * p <0.05, ** p < 0.01, *** p < 0.001.
